# The Effectiveness of a Home Care Program for Supporting Caregivers of Persons with Dementia in Developing Countries: A Randomised Controlled Trial from Goa, India

**DOI:** 10.1371/journal.pone.0002333

**Published:** 2008-06-04

**Authors:** Amit Dias, Michael E. Dewey, Jean D'Souza, Rajesh Dhume, Dilip D. Motghare, K. S. Shaji, Rajiv Menon, Martin Prince, Vikram Patel

**Affiliations:** 1 Department of Preventive and Social Medicine, Goa Medical College, Goa, India; 2 Section of Epidemiology, Institute of Psychiatry, King's College, London, United Kingdom; 3 Dementia Society of Goa, Goa, India; 4 Directorate of Health Services, Goa, India; 5 Department of Psychiatry, Medical College, Thrissur, India; 6 Mental Health Centre, Chelsea & Westminster Hospital, London, United Kingdom; 7 Department of Epidemiology and Population Health, London School of Hygiene and Tropical Medicine, London, United Kingdom; University of Cambridge, United Kingdom

## Abstract

**Objectives:**

To develop and evaluate the effectiveness of a home based intervention in reducing caregiver burden, promoting caregiver mental health and reducing behavioural problems in elderly persons with dementia.

**Methodology and Principal Findings:**

This was a randomised controlled trial in which the person with dementia-caregiver dyad was randomly allocated either to receive the intervention immediately or to a waiting list group which received the intervention after 6 months. It was carried out in communities based in two talukas (administrative blocks) in Goa, India. Mild to moderate cases with dementia (diagnosed using the DSM IV criteria and graded using the Clinical Dementia Rating scale) and their caregivers were included in the trial. Community based intervention provided by a team consisting of Home Care Advisors who were supervised by a counselor and a psychiatrist, focusing on supporting the caregiver through information on dementia, guidance on behaviour management, a single psychiatric assessment and psychotropic medication if needed. We measured caregiver mental health (General Health Questionnaire), caregiver burden (Zarit Burden Score), distress due to behavioural disturbances (NPI-D), behavioural problems in the subject (NPI-S) and activities of daily living in the elder with dementia (EASI). Outcome evaluations were masked to the allocation status. We analysed each outcome with a mixed effects model. 81 families enrolled in the trial; 41 were randomly allocated to the intervention. 59 completed the trial and 18 died during the trial. The intervention led to a significant reduction of GHQ (−1.12, 95% CI −2.07 to −0.17) and NPI-D scores (−1.96, 95%CI −3.51 to −0.41) and non-significant reductions in the ZBS, EASI and NPI-S scores. We also observed a non-significant reduction in the total number of deaths in people with dementia in the intervention arm (OR 0.34, 95% CI 0.01 to 1.03).

**Conclusion:**

Home based support for caregivers of persons with dementia, which emphasizes the use of locally available, low-cost human resources, is feasible, acceptable and leads to significant improvements in caregiver mental health and burden of caring.

ClinicalTrials.gov NCT00479271

## Introduction

Recent estimates show that dementia is a major cause of burden of disease amongst the elderly in developing countries[1]. As many parts of the developing world witness a demographic transition, dementia is likely to account for an even greater proportion of this burden in the future[2]. The numbers of people affected by dementia in India are set to triple, reaching six million by 2040[3]. Awareness is limited, both in the community and among health professionals[4] and there are few services tailored to the needs of people with dementia and their caregivers.

Service development for older people with dementia in developing countries needs to take account of the prevailing socio-economic, health system and cultural circumstances. Dementia is generally perceived to be part of normal ageing, and families rarely present to health services[4]. Health services are ill-equipped to meet the needs of older persons. Health care is typically clinic-based; the person with dementia and their caregivers must attend a clinic or hospital, often involving a long journey and waiting time. The assessment and treatment that they receive is orientated towards acute rather than chronic conditions. As a consequence, most care for dementia is informal, with little or no support from health or social services[5]. Our work in India has shown that caring is associated with markedly worse mental health, higher caregiver burden, and greater out of pocket health care costs due to reliance on private doctors who are able to make home visits[6].

The 10/66 Dementia Research group has proposed that existing or locally available healthcare resources be mobilized to provide outreach needs assessments and continuing care[1]. Our home based intervention for people with dementia and their families in Goa, India is inspired by this model. We have sought, using the 10/66 outcome measures, to evaluate a demonstration project testing the effectiveness of the intervention in reducing caregiver burden, promoting caregiver mental health, and reducing behaviour problems in elderly persons with dementia.

## Methods

The protocol for this trial and supporting CONSORT checklist are available as supporting information; see CHECKLIST S1 PROTOCOL S1.

**Setting**: The study was conducted in Goa, on the west coast of India. Goa is in an advanced stage of the epidemiological and demographic transition in the country, with some of the best health indicators [7] and a rising proportion of older people; the 2001 Census reported that 8.3% of the total population was aged over 60 years[8]. The consensus figure for prevalence of dementia in India is 1.9% above the age of 60 years [3]The present trial was carried out in two of the largest administrative blocks (talukas) in the state - Bardez and Tiswadi with a population of approximately 340,000.

**Recruitment:** Information about dementia was widely disseminated through handouts, newspaper articles and through private and public health services. Concerned relatives and older people were urged to contact a special help line. Probable cases of dementia were also identified with the help of key informants (doctors, priests, health workers, local leaders). All probable cases were examined by a trained clinician (AD) to confirm the diagnosis of dementia according to DSM IV criteria[9] and graded using the Clinical Dementia Rating (CDR) Scale[10]. Our inclusion criteria were: CDR mild and moderate dementia. Exclusion criteria were: CDR severe dementia or severe co-morbid physical health conditions. The principal caregiver, as identified by the family, was enrolled for the trial. The principal caregiver was generally the spouse, although in some instances another family member was the principal caregiver, particularly when the spouse was not in a position to care.

**Measures at Recruitment:** Baseline information was collected from the principal caregiver by two trained field researchers before randomization. The researchers underwent an intensive one week training in interview techniques with role-play to familiarize them with the instruments and ensure inter-observer reliability. and used standardized questionnaires for collecting baseline information, as follows:

- Socio demographic characteristics of the person with dementia and the caregiver
- Everyday Abilities Scale for India (EASI): This questionnaire consisting of 12 questions, has been developed and widely used to test the functional abilities of daily living relevant to Indian subjects[11]
- Neuro-Psychiatric Inventory (NPI) Questionnaire: This instrument consists of two parts; the first measures the severity of the problem behaviours associated with the condition on a scale of 1–3 (NPI- S); the second measures the perceived distress of the problem behaviours by the caregiver on a scale of 0–5 (NPI -D)[12].
- Zarit Burden scale (ZBS): This is the most widely used scale in the studies of caregiver burden and encompasses the physical, emotional and financial burden as perceived by the caregiver[13].
- General Health Questionnaire (GHQ): The 12 question GHQ is used to measure the psychological impact on the caregivers' mental health.

All these instruments were translated into Konkani, the local language of Goa, using standard methods of translation and back-translation. These instruments have been used in India for the 10/66 caregiver studies[6].

**Randomization:** Randomization of dyads comprising the person with dementia and their principal caregiver was carried out by an independent person, based on simple random number tables, either to the intervention or waiting list group (who received the intervention after six months).

**Intervention:** The principles of the intervention were that, first, it had to utilise locally available health and human resources so that there was a good probability that it might be affordable for scaling up; and second, that it needed to be community and home-based, since many people with dementia and their families had difficulties accessing public health services. The intervention was a flexible, stepped-care model primarily aimed to improve the awareness and knowledge of family caregivers regarding dementia, to provide emotional support to caregivers, to maximise their caregiving resources and to improve their caregiving skills.

The intervention was delivered by a Community Team, one for each taluka. Each team comprised two full-time Home Care Advisors (HCA), and a part-time local psychiatrist from the public health services, and a part-time lay counselor (who was shared by both teams). The minimum requirements for being a HCA were knowledge of the local language, being literate, preferably passed higher secondary school, and motivated to be involved in the community care of older people. They received intensive training for a week through role play and interactive training methods. The HCA were trained in key skills including listening and counseling skills, bereavement counseling, stress management and health advice for common health problems. The specific components of the intervention carried out by the HCA were:

- Basic education about dementia (what is the disease, its course, its features etc)
- Education about common behaviour problems and how they can be managed
- Support to the caregiver, for example for an elderly caregiver living alone with the patient, in activities of daily living
- Referral to psychiatrists or the family doctor when behaviour problems are severe and warrant medication intervention.
- Networking of families to enable the formation of support groups.
- Advice regarding existing government schemes for elders

The HCA applied a flexible home-care program tailored to the needs of the individual and the family. The baseline information collected by the researchers was made available to the HCA before they initiated the intervention. The minimum frequency of visits was at least once a fortnight for six months. The maximum was based on the needs as assessed by the HCA. Thus, the visits could be more frequent depending on the need of that particular family.

The HCA were supported, and supervised, by the two part-time specialists: two psychiatrists (one supporting each team) and one counsellor (supporting both teams). Each person with dementia was seen at least once by a local psychiatrist who advised regarding use of medication for behaviour and other common medical problems based on an agreed protocol. The caregiver and the person with dementia were encouraged to visit the psychiatrist in the clinic so that, if medication or clinical investigations were needed, these could be availed of at no cost from the public health service, and because the time required for travel for the psychiatrist for home visits was considered to be a precious resource. A home visit was arranged if a clinic visit was not possible. HCA would meet the psychiatrist twice a month and update them on the progress of the person with dementia, particularly those who were receiving medication. The other specialist was a lay counselor (JD) who had herself been a caregiver for a parent with dementia. The HCA from both talukas met with the counsellor once a fortnight to share experiences, support one another, and problem solve difficult situations.

**Control arm:** The control arm dyads received only education and information regarding dementia and were then placed in a waiting list to receive the intervention after 6 months. They were free to utilize the existing health services during this time.

**Outcomes**: Outcome assessments were carried out at 3 and 6 months using the same instruments as in the baseline interview. Outcome evaluations were carried out by researchers who were masked to the allocation status until the end of the project. We attempted to blind outcome evaluations by ensuring that allocation status was kept in a separate office from the outcome evaluation teams. We had also instructed the families not to divulge information on the visits by the Home Care Advisor. However, we anticipated that some unmasking would occur because both the intervention and outcome evaluations were home-based. In order to evaluate the masking process, researchers were asked to guess the intervention status. Our primary outcome was the caregiver mental health (GHQ score). Secondary outcomes were perceived burden (Zarit Burden score), distress due to problem behaviours (NPI-D) and severity of the behavioural problems in the person with dementia (NPI-S), and functional ability of the subject (EASI). Death records were collected for all people with dementia who died during the course of the project and a caregiver GHQ was carried out one month after the death.

**Analysis:** We analysed each of the outcome measures (GHQ, Zarit, EAS, NPI-S and NPI-D) with a mixed effects model. The basic model included time with two levels (3 and 6 months), treatment and the baseline score as covariates, and a random intercept. We coded time to start from the 3 month time point so that in the event of any interaction with time the treatment effect would represent the effect at 3 months. We also fitted an additional model with time as a random effect and a model including the time by treatment interaction. Mixed effects modelling has the benefit of using all the available data. As a secondary analysis we also repeated the model for GHQ with the addition of a further fixed effect for pattern of missing values. This had three levels: person with dementia died between baseline and 3 months, died between 3 and 6, and still alive at 6 months. The decision to include only the outcome GHQ in this secondary analysis was made jointly by three of the authors (AD, VP, MP) masked to the outcome of the analysis. We examined residuals to check for violation of assumptions. We fitted the models using the lme package in R [14] and present the estimated coefficients with 95% confidence intervals. We modelled the effect of treatment on mortality using a logistic regression with treatment, age and sex as covariates. We present the estimated odds ratio with a likelihood-based 95% confidence interval.

**Ethical Considerations:** The proposal was approved by the Dementia Society of Goa ethics committee. Caregivers were recruited only after written informed consent to participate in the trial was obtained from them. We also obtained a verbal assent from the person with dementia in the presence of the caregiver and the same was recorded. This procedure was approved by the ethics committee. The head of the family (if other than the caregiver) was also informed about the nature of the trial and intervention. We did not take a written consent from the people with dementia because the intervention was focused on, and research interviews were only carried out with, the caregiver. There was no restriction on usual care arrangements, and ultimately, the caregivers of the people with dementia in the waiting list group were also provided the intervention.

## Results

We had originally set a sample of 80 persons with dementia, based on our estimates of how many individuals we were likely to be able to enrol given the time and human resources at our disposal and the geographical area of coverage of the program. Finally eighty one people with dementia and their principal caregivers were enrolled in the trial. The baseline characteristics with regards to the socio-demographic profile of the intervention and control groups were similar (Table 1).There were no baseline differences in socioeconomic status and psychiatric co-morbidity. Outcome measures at baseline were also similar except for the mean GHQ scores, which were higher in the intervention group (Table 2). This difference was adjusted for in subsequent analyses. Seventy (86%) of the principal caregivers lived with the person with dementia, the majority (58%) having no help from any other relative for their caregiving activities. Forty one (50.6%) families recruited in the study had accessed health care for the person with dementia, in the three months prior to recruitment; of those seeking help, five attended primary care, six used a hospital service and 30 used a private doctor who would visit the home to examine the person with dementia when required.

**Table 1:** Baseline characteristics of the sample of dyads of persons with dementia and caregivers in a RCT of a community intervention for dementia in Goa, India

Group status			Intervention N = 41	Control N = 40	
**Age**	Person with dementia	Mean	79.4	77.3	F = 1.4,df = 1,p = 0.2
		SD	8	8	
	Caregiver	Mean	53.2	53.8	F = 0.01,df = 1,p = 0.9
		SD	14	16	
**Gender**	Person with dementia	Males	26 (63.4)	27 (67.5)	Chi^2^ = 0.14,df = 1,p = 0.4
	Caregiver	Males	4 (9.8)	6 (15)	Chi^2^ = 5.1,df = 1,p = 0.4
**Marital status**	Person with dementia	Currently married	14 (34.1)	17 (42.5)	Chi^2^ = 0.6,df = 1,p = 0.3
	Caregiver	Currently married	31 (75.6)	36 (90)	Chi^2^ = 4.1,df = 1,p = 0.2
**Relationship to subject**		Spouse	12 (29.3)	15 (37.5)	Chi^2^ = 3.2,df = 1,p = 0.8
**Education**	Person with dementia	Below primary	16 (39)	19 (47.5)	Chi^2^ = 2.6,df = 1,p = 0.8
	Caregiver	Below primary	8 (19.5)	9 (22.5)	Chi^2^ = 3.6,df = 1,p = 0.5
Availability of Paid help	Day time		5 (12. 2)	6 (15.0)	Chi^2^ = 0.1,df = 1,p = 0.96
	Night time		3 (7.3)	4 (10.0)	F = 0.18,df = 1,p = 0.71
Per capita monthly Income(Indian rupees)	Mean		1209	1768	t = 1.38, p = 0.2
	SD		1353 (100–5000)	2435(200–13333)	

Figures in parenthesis indicate percentages

**Table 2:** Outcome measures at baseline and review in a sample of dyads of persons with dementia and caregivers in a RCT of a community intervention for dementia in Goa, India

Outcome measure		EASI		ZBS		NPIQ-S		NPIQ-D		GHQ	
		Intervention	Control	Intervention	Control	Intervention	Control	Intervention	Control	Intervention	Control
Baseline	Mean	8.3	8.3	24.8	21.7	10.2	10	8.6	9.2	4	2.5
	N	41	40	41	40	41	40	41	40	41	40
	SD	2.6	2.7	14.9	13.1	5.8	6.1	6.9	8.3	2.8	2.3
3 months	Mean	8.4	9.1	19.5	21.5	6.4	8	3.8	6.9	3.1	2.9
	N	34	32	34	32	34	32	34	32	34	37
	SD	2.5	2.1	13	17.1	4.6	4.9	4.3	5.3	4.3	3.3
6 months	Mean	8.5	8.7	19	21.4	6.7	8.4	4.4	7.1	2.6	3.3
	N	33	26	33	26	33	26	33	26	34	31
	SD	2.3	2.2	13	16.2	4.8	5.1	3.8	6.4	2.3	3.6

**Figure 1:**
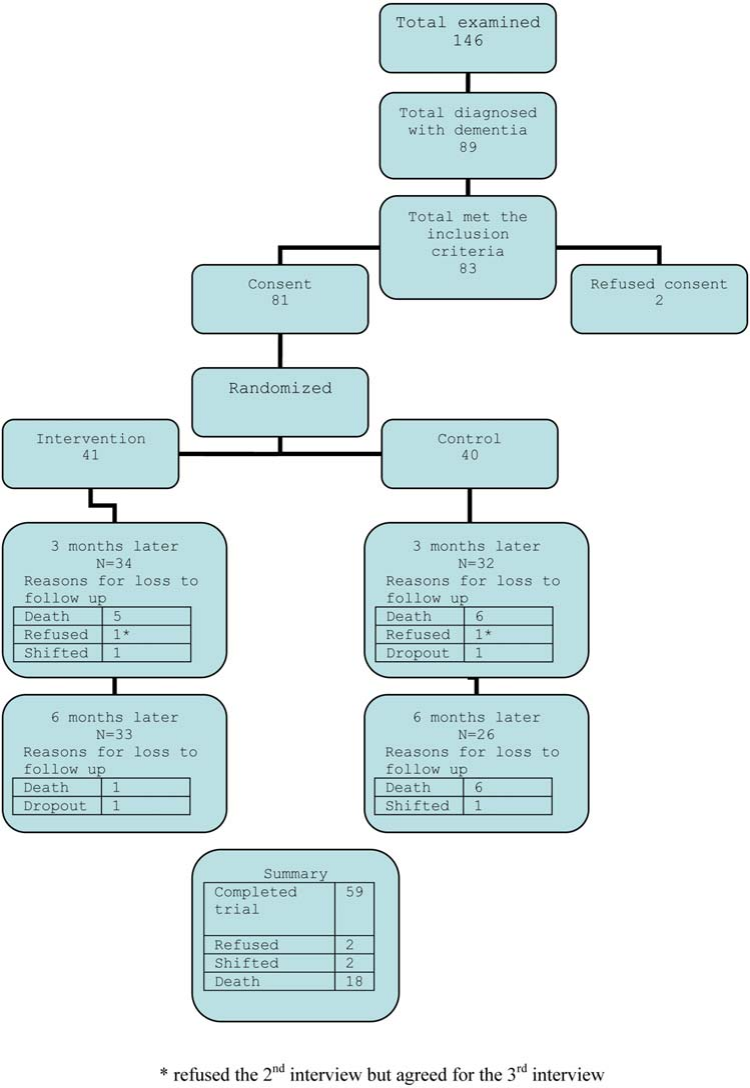
Dementia Trial Flow Chart

Mortality was high; 22% (18) of the people with dementia died during the trial review period. The commonest causes of death were stroke (n = 4), pneumonia (n = 4), myocardial infarction (n = 3), and septicaemia (n = 2). Two families moved out of the study area and two refused to continue with the trial. The trial flow chart is shown in Figure 1. There was no significant difference in the baseline characteristics of those who died or were alive till the end of the trial (p>0.05 for GHQ, NPI-S, NPI-D, EASI, ZBS scores).

In the intervention arm, the mean number of visits by the home care advisor was 12.3 (SD = 3.1). Average time spent on each visit was 45 minutes (SD = 15). The mean number of phone consultations were 1.3 (SD = 2.1). A total of nine support group meetings were arranged for the caregivers during the intervention period. Nineteen caregivers could not attend the meetings. The most common reasons cited were lack of transport-53% (10), no one to look after the person with dementia-26% (5), and not wanting to make a public appearance- 16%(3). All the families received at least one visit by, or to, a psychiatrist. Although we encouraged the family to visit the psychiatrist at the clinic, psychiatrists were still needed to carry out 21 home visits. Four of the subjects were receiving anticholinesterases before the start of the intervention. Psychiatrists prescribed anticholinesterases to nine other individuals; however only three agreed to commence these drugs. The reasons for refusing medication were that they could not afford medicines, the family doctor thought it was unnecessary or they felt it would have side effects. Ten persons with dementia were receiving anti-depressants before the intervention; the study psychiatrists advised 2 more patients such medications. These numbers were too small for us to investigate the specific effect of these treatments on the outcomes. The researchers could correctly guess the intervention status in 65.4% (53) of the families who enrolled.

There was no need for a random effect of time or for an interaction between time and treatment for any of the five outcomes. Table 3 shows the coefficients from the models and their 95% confidence intervals. The treatment significantly affected both GHQ and NPI distress leading in both cases to a net reduction of slightly more that 1 point for GHQ and almost 2 points for NPI distress. There was no significant effect of time for other outcomes. Residual plots revealed no problems with the fitted models. In the secondary analysis of GHQ, adding a further variable for pattern of dropout due to death, the conclusions remain unchanged. The treatment lowered the risk of death during the 6 month period. However this reduction was not statistically significant (odds ratio 0.34, 95% confidence interval 0.01 to 1.03).

**Table 3:** Coefficient (95% confidence intervals) from the mixed effects models

	Treatment			Time		
Outcome	Effect	95% CI		Effect	95% CI	
GHQ	−1.12	−2.07	−0.17	0.03	−0.14	0.19
Zarit	−3.29	−6.78	0.20	−0.12	−0.99	0.73
EAS	−0.36	−1.02	0.31	−0.03	−0.14	0.09
NPI severity	−1.19	−2.83	0.46	0.11	−0.25	0.47
NPI distress	−1.96	−3.51	−0.41	0.09	−0.36	0.54

## Discussion

Caregiver focused interventions for persons affected by dementia have previously only been described in developed countries[15]. This paper presents the results of the first trial evaluating the effectiveness of a community based intervention for persons with dementia and their caregivers. Our main findings are that the intervention led to significant improvements in caregiver mental health and perceived burden; non-significant reductions were observed for behaviour disturbances and functional ability. Overall, we observed a high mortality in our cohort of persons with dementia, and a 64% reduction in the risk of death in the intervention arm. This reduction, however, was not statistically significant.

The principles underlying our intervention was that it had to be community based since many patients with dementia and their caregivers were unable to attend health facilities due to mobility difficulties and lack of transport. The intervention had to be sustainable, i.e. relying mostly on existing health resources or low-cost additional resources; thus, the front line of our intervention was a locally recruited individual who had no prior experience with dementia care and was not a health professional; training was carried out using local materials and resource persons; and the support and supervision was provided by local psychiatrists and counsellors. This model ensured that more services (for example, number of visits or medication) were provided to those who were in greater need. The intervention in this study was modelled on the work of authors in developed countries [15], [16]and modified to suit the local health system realities of our study setting. It included both pharmacological and psychosocial interventions to improve the health needs of the caregiver and the person with dementia in their home.

An unexpected finding of our study was the high mortality of patients, despite the fact that we excluded subjects with severe dementia. We also observed a non significant reduction in mortality in the intervention arm. Poor quality of care due to a low degree of awareness of the disorder, and poor management of complications such as nutritional deficiencies and vascular events, could be responsible for the large number of deaths during the study period. We speculate that the provision of a medical assessment and consequent treatments for common medical complications, the improvement in caregiver mental health, and better information regarding caring for the person with dementia, may all have contributed to the reduced mortality. Similar findings suggesting higher survival rates for persons with dementia receiving home-based care interventions have been reported from richer countries[15].

The finding that our intervention improved caregiver mental health, but did not have a significant impact on behaviour of the person with dementia echoes the findings of some other studies. McCurry et al (1998) showed that their behavioural intervention was successful in improving the sleep of caregivers but was not successful in improving caregiver burden or patient problem behaviours[17]. Hinchliffe et al (1995) on the other hand showed that a multidisciplinary team approach, combining medication, psychological techniques and social measures, showed a significant improvement in caregiver mental health and problem behaviours of the person with dementia[18]. In a relatively large study, Mittleman et al [16], [19], [20]showed that an intervention involving family counselling and support group meetings reduced nursing home placements by half and significantly improved caregiver mental health within one year. Nursing home placement, as an outcome measure, could not be studied in the present trial as the aged homes in Goa do not admit people with dementia and there are no specialised dementia homes in the State. A study conducted in Finland showed that an intervention involving home visits, counselling, follow up calls, social and health care services, did not show any significant difference in the number of patients having moved to long-term care nor did it show a difference in the deaths[21].

The key limitation of our trial is the relatively small sample size which was probably inadequately powered to detect significant reductions in behaviour problems and functional abilities. Furthermore, we only followed patients for six months, in part because we anticipated high mortality (an assumption which was confirmed). The short follow up period may have precluded us demonstrating effects. Since dementia is a chronic progressive disease, services based on our intervention model would have to be an ongoing process throughout the life of the person with dementia. We accept that while snowballing may not lead to a genuinely representative sample, it remains the most pragmatic and cost-effective method for case-detection in low resource settings [22]. Another limitation in trials of this nature is that the researchers did, during the course of their outcome evaluation, correctly guess the allocation status in nearly two-thirds of individuals because of the information on health care use which typically led some care-givers to share contacts with the intervention team. There were no protocol violations. In two cases there were objections to the intervention by the HCA by a non-resident family member while in another instance, there were objections raised by the General Practitioner to the drugs prescribed by the psychiatrist. However they agreed to the intervention after we explained the programme to them. We were able to achieve a high follow up rate in our cohorts with only 5% of families dropping out of the study.

In conclusion, our pilot trial shows that a community based intervention using locally available resources is feasible, acceptable and leads to significant improvements in caregiver mental health and burden of caring and is associated with reduced mortality of the person with dementia. Larger trials are needed to demonstrate the effect of such an intervention with greater confidence.

In the context of the rising burden of dementia in developing countries which are witnessing a demographic transition, such community based interventions have considerable potential to improve the quality of life of the caregiver and the person with dementia. Future research should evaluate the effectiveness of a similar intervention, utilizing community health workers to identify cases of dementia[22], [23] and replacing the psychiatrist (a scarce resource in many developing countries) with a general practitioner trained in the management of dementia.

## Supporting Information

Checklist S1 CONSORT Checklist.
(0.06 MB DOC)

Protocol S1 Trial Protocol.
(0.07 MB DOC)
