# Supplementary material for: The Effectiveness of a Home Care Program for Supporting Caregivers of Persons with Dementia in Developing Countries: A Randomised Controlled Trial from Goa, India
Source: PLoS One. 2008 Jun 4;3(6):e2333. doi: 10.1371/journal.pone.0002333 (PMC2396286; doi:10.1371/journal.pone.0002333)
Supplement: Checklist S1 — CONSORT Checklist. (0.06 MB DOC) [file pone.0002333.s001.doc]

**CONSORT CHECK LIST**

Manuscript Title:

**The Effectiveness Of A Home Care Program For Supporting Caregivers Of Persons With Dementia In Developing Countries: A randomised controlled trial from Goa, India**

First Author:

Dr. Amit Dias, MD, DTM&H, DGM,

Epidemiologist and Geriatrician,

Coordinator, 10/66 Dementia Research Group

Lecturer, Dept. of Preventive and Social Medicine,

Goa Medical College.

Bambolim Goa India. 403202

Ph: +91-832-2414027, E mail: [dr_amit_dias@yahoo.com](mailto:dr_amit_dias@yahoo.com)

| Heading | Subheading | Descriptor | Was it reported? Yes or No | If "Yes", what section? |
| --- | --- | --- | --- | --- |
| Title |  | 1. Identify the study as a randomised trial | YES | title |
| Abstract |  | 2. Use a structured format | YES | abstract |
| Introduction |  | 3. State prospectively defined hypothesis, clinical objectives, and planned subgroup or covariate analyses. | YES | Introduction |
| Methods | Protocol | Describe |  |  |
|  |  | 4. Planned study population, together with inclusion/ exclusion criteria | YES | Method |
|  |  | 5. Planned interventions and their timing | YES | Method, Intervention |
|  |  | 6. Primary and secondary outcome measure(s) and the minimum important difference(s),  ….and how the target sample size was projected. | YES  YES | Outcome  Results |
|  |  | 7. Rationale and methods for statistical analyses, detailing main comparative analyses and whether they were completed on an intention-to-treat basis | YES | Analysis |
|  |  | 8. Prospectively defined stopping rules (if warranted). | None |  |
|  | Assignment | Describe |  |  |
|  |  | 9. Unit of randomisation (eg individual, cluster, geographic). | YES | Method, Randomisation |
|  |  | 10. Method used to generate the allocation schedule. | YES | Method |
|  |  | 11. Method of allocation concealment and timing of assignment. | YES | Method |
|  |  | 12. Method to separate the generator from the executor of assignment | YES | Method |
|  | Masking (Blinding) | 13. Describe mechanism (eg, capsules, tablets); similarity of treatment characteristics (eg appearance, taste); allocation schedule control (location of code during trial and when broken); and evidence for successful masking  (blinding) among participants, person doing intervention, outcome assessors, and data analysts. | YES  (No Placebo involved in this study. Evaluators were masked to intervention status) | Outcome/ Analysis |
| Results | Participant Flow and follow- up | 14. Provide a trial profile as a Figure in the manuscript (see flow diagram summarizing participant flow, numbers and timing of randomization assignment, interventions, and measurements for each randomised group) | YES | Trial Flow Chart |
|  | Analysis | 15. State estimated effect of intervention on primary and secondary outcome measures, including a point estimate and measure of precision (confidence interval). | YES | Results/ Table 2 |
|  |  | 16. State results in absolute numbers when feasible (eg 10/20, not 50%) | YES | Results, discussion, figure 1, Table 1,2,3. |
|  |  | 17. Present summary data and appropriate descriptive and inferential statistics in sufficient detail to permit alternative analyses and replication. | YES | Results, discussion, figure 1, Table 1,2,3. |
|  |  | 18. Describe prognostic variables by treatment group and any attempt to adjust for them. | YES | Results/ Table 2 |
|  |  | 19. Describe protocol deviations from the study as planned, together with the reasons. | (No deviations) |  |
|  | Discussion | 20. State specific interpretation of study findings, including a discussion of internal bias (the degree to which the trial design, conduct analysis and presentation have minimised or avoided biased comparisons of the interventions under evaluation) and external bias (the precision and extent to which it is possible to generalise the results of the trial to other settings) | YES | Discussion |
|  |  | 21. State general interpretation of the data in light of the totality of the available evidence. | YES | Discussion |
