# Supplementary material for: The Effectiveness of a Home Care Program for Supporting Caregivers of Persons with Dementia in Developing Countries: A Randomised Controlled Trial from Goa, India
Source: PLoS One. 2008 Jun 4;3(6):e2333. doi: 10.1371/journal.pone.0002333 (PMC2396286; doi:10.1371/journal.pone.0002333)
Supplement: Protocol S1 — Trial Protocol. (0.07 MB DOC) [file pone.0002333.s002.doc]

## The Dementia Home Care Project

Trial Protocol

|  |  |
| --- | --- |

| **Title** | The Dementia Home Care Project: Evaluating the effectiveness of a community based, flexible, stepped care intervention for persons with dementia and their caregivers in a developing country |
| --- | --- |
| **Summary** | The aim of this trial is to evaluate a home based, flexible, stepped-care intervention designed to improve the awareness and knowledge of family caregivers regarding dementia, to maximise their caregiving resources, and to improve their caregiving skills. |
| **Study Design** | Randomized controlled trial  Single-blind (outcome evaluations blinded) |
| **Description** | Service development for older people with dementia in India needs to take account of the fact that dementia tends to be a hidden problem. Thus, dementia is generally perceived to be part of normal ageing, and not a health condition, and families rarely present to health services, who are generally ignorant of most cases in their community. Health services are often ill-equipped to meet the needs of older persons. Health care, even primary care, is clinic-based; the older person must attend the clinic or hospital, often involving a long journey and waiting time. The assessment and treatment that they receive is orientated towards acute rather than chronic conditions. Thus, home-based interventions provide a practical alternative to clinic-based interventions for persons affected by dementia.  **Objectives:** The project has two specific objectives:   - To develop and apply a flexible, stepped-care intervention designed to improve the awareness and knowledge of family caregivers regarding dementia, to maximise their caregiving resources and to improve their caregiving skills. - To evaluate the efficacy and cost-effectiveness of the intervention in reducing carer burden and improving carer mental health, reducing behaviour problems in elderly persons with dementia and reducing the costs of illness.   **Sample:** Probable cases of dementia will be identified with the help of key informants (doctors, priests, health workers, local leaders) in two of the most populated *talukas* (counties) of Goa (Bardez and Tiswadi,). All probable cases were examined by a trained clinician (AD) to confirm the diagnosis of dementia according to DSM IV criteria and graded using the Clinical Dementia Rating (CDR) Scale. Our inclusion criteria are: CDR mild and moderate dementia. Exclusion criteria str: CDR severe dementia or severe co-morbid physical health conditions. The principal caregiver, as identified by the family, will be enrolled for the trial.  Cases will be randomized to receive the intervention or placed in a waiting list group who will receive the intervention after 6 months.  **Randomisation and Intervention:** All eligible subjects will be allocated a study number by a secretary, and the study numbers randomised into two groups by an independent person. This person involved with the process of randomization will be blind to the identity of the subject. A random number table will be used for the process of randomization. The person allocating the study number will inform the secretary of the allocation status; the secretary will then inform the concerned Home Care Advisor (HCA-see below for description of intervention) if the study number has been allocated to receive the intervention. The HCA will then be contact the family allotted to them.  **Baseline interview:** The baseline assessment will assess the demographic characteristics of the family, the background information about the principal carer and the subject, costs of caring for the person with dementia, behavioural problems in the subject, perceived burden and the impact on the carers mental health. This information will be made available to the HCAs before they initiate the intervention.  **Intervention:** The intervention is designed to provide family caregivers with information about dementia syndrome, assistance in setting up local support groups with other caregivers, and training in strategies to better manage common behavioural problems in the home setting. This package would form the basis of the community intervention to be delivered in the proposed project by a Home Care Advisor (HCA) who has been recruited by the community and trained in the delivery of the package. Some patients with dementia suffer from behavioural or psychiatric problems which may improve considerably with the judicious use of psychotropic medication. The use of such medication requires the assessment and evaluation by a physician who has had basic training in the use of sedative medication in elderly people. Such a referral system will be made available through the Home Care Advisor.  Following suitable training, the Home Care Advisors will apply a flexible home-care program tailored to the needs of the individual and the family. The components of the intervention will include:   - Basic education about dementia (what is the disease, its course, its features etc) - Education about common behaviour problems and how they can be managed - Support to the carer, for example for an elderly carer living alone with the patient, in activities of daily living - Referral to specialists when behaviour problems are severe and warrant medication intervention (sedatives).   **Control arm:** These subjects would receive information/education about dementia and be placed on a waiting list to receive the full intervention after 6 months. The comparison of the two arms in terms of specific intervention components is shown in the table below.   | Intervention | Intervention group | Control group | | --- | --- | --- | | Education/ Information | + | + | | Intervention by Home Care Advisors | + | - | | Medical assessment and treatment if necessary* | + | - | | Unrestricted Medical Care  *( no one will stop the subjects from taking medical help)* | + | + | | Networking  *Support groups* | + | - |   **Outcome evaluation:** Outcomes will be assessed at 3 and 6 months after enrolment and will include:   - Everyday Abilities Scale for India (EASI): This questionnaire consisting of 12 questions, has been developed and widely used to test the functional abilities of daily living relevant to Indian subjects - Neuro-Psychiatric Inventory (NPI) Questionnaire: This instrument consists of two parts; the first measures the severity of the problem behaviours associated with the condition on a scale of 1-3 (NPI- S); the second measures the perceived distress of the problem behaviours by the caregiver on a scale of 0-5 (NPI -D). - Zarit Burden scale (ZBS): This is the most widely used scale in the studies of caregiver burden and encompasses the physical, emotional and financial burden as perceived by the caregiver - General Health Questionnaire (GHQ): The 12 question GHQ is used to measure the psychological impact on the caregivers’ mental health. |
| **Primary Outcome Measure(s)** | 1. Carer mental health 2. Carer burden 3. Behaviour problems and ADL in elderly persons with dementia 4. Costs of illness. |
| **Secondary Outcome Measure(s)** | Process indicators: average number of visits by Home Care Advisor, visits by Psychiatrists, use of medication  Effect of intervention on survival and overall quality of life of the person with dementia as well as the carer |
| **Sample Size** | Since this is the first trial of its kind in a developing country, we based the total number of subjects on studies conducted in the developed world which averaged around 40 in each arm. No a priori sample size estimation was carried out. |
| **Setting** | Bardez and Tiswadi – two of the largest talukas (administrative counties) in the State of Goa |
| **Project Coordinator** | Dr. Amit Dias, MD, DTM&H (UK), DGM,  Project Coordinator; Epidemiologist & Geriatrician, Lecturer,  Dept. of Preventive and Social Medicine,  Goa Medical College. &  Secretary, The Dementia Society of Goa.  Jt. Secretary, Alzheimer’s and Related Disorders Society of India.  Member, 10/66 dementia research group  Mob: 9822382842 Res: 2414027  E mail: [apdias@sancharnet.in](mailto:apdias@sancharnet.in) |
| **Overall Study Official(s)** | Dr Vikram Patel (Principal Investigator)  Dr. Amit Dias (Project Coordinator) |
| **Human Subjects Review/Oversight** | Ethical IRB approvals obtained from the Ethics Committee of the Dementia Society of Goa |
| **Study Sponsor** | World Health Organization through the Ministry of Social Justice and Empowerment, Government of India |
| **Collaborators** | Institutions:   1. The Dementia Society of Goa 2. Department of Preventive and Social Medicine, Goa Medical College 3. London School of Hygiene & Tropical Medicine   Individuals   1. Mr. Michael Dewey, Senior Lecturer in Medical Statistics, Section of Epidemiology, King's College, London, Institute of Psychiatry, UK 2. Mrs. Jean D’Souza, Senior Counselor, Dementia Society of Goa, India 3. Dr. Rajesh Dhume, Consultant Psychiatrist, Directorate of Health Services, Goa, India 4. Prof. D.D. Motghare, Professor and Head, Dept. of Preventive and Social Medicine, Goa Medical College, India. 5. Dr. K. S. Shaji, Assistant Professor of Psychiatry, Medical College, Thrissur, India. 6. Dr. Rajiv Menon, Consultant Psychiatrist for Older Adults, Mental Health Centre, Chelsea & Westminster Hospital, UK 7. Dr. Martin Prince, Professor, Section of Epidemiology, King's College, London, Institute of Psychiatry, UK |
| **Funding Source** | World Health Organisation |
